# Differential expression of cell cycle regulators in CDK5-dependent medullary thyroid carcinoma tumorigenesis

**DOI:** 10.18632/oncotarget.3813

**Published:** 2015-04-14

**Authors:** Karine Pozo, Antje Hillmann, Alexander Augustyn, Florian Plattner, Tao Hai, Tanvir Singh, Saleh Ramezani, Xiankai Sun, Roswitha Pfragner, John D. Minna, Gilbert J. Cote, Herbert Chen, James A. Bibb, Fiemu E. Nwariaku

**Affiliations:** ^1^ Department of Psychiatry, The University of Texas Southwestern Medical Center, Dallas, TX, USA; ^2^ Hamon Center for Therapeutic Oncology Research, The University of Texas Southwestern Medical Center, Dallas, TX, USA; ^3^ Harold C. Simmons Comprehensive Cancer Center, The University of Texas Southwestern Medical Center, Dallas, TX, USA; ^4^ Department of Endocrine Neoplasia and Hormonal Disorders, The University of Texas MD Anderson Cancer Center, Houston, TX, USA; ^5^ Department of Radiology, The University of Texas Southwestern Medical Center, Dallas, TX, USA; ^6^ Institute of Pathophysiology and Immunology, Medical University of Graz, Graz, Austria; ^7^ Department of Pharmacology, The University of Texas Southwestern Medical Center, Dallas, TX, USA; ^8^ Endocrine Surgery Research Laboratory, The University of Wisconsin Carbone Cancer Center, Madison, WI, USA; ^9^ Department of Neurology and Neurotherapeutics, The University of Texas Southwestern Medical Center, Dallas, TX, USA; ^10^ Department of Surgery, The University of Texas Southwestern Medical Center, Dallas, TX, USA

**Keywords:** Cdk5, retinoblastoma protein, neuroendocrine, medullary thyroid carcinoma, cell cycle

## Abstract

Medullary thyroid carcinoma (MTC) is a neuroendocrine cancer of thyroid C-cells, for which few treatment options are available. We have recently reported a role for cyclin-dependent kinase 5 (CDK5) in MTC pathogenesis. We have generated a mouse model, in which MTC proliferation is induced upon conditional overexpression of the CDK5 activator, p25, in C-cells, and arrested by interrupting p25 overexpression. Here, we identify genes and proteins that are differentially expressed in proliferating versus arrested benign mouse MTC. We find that downstream target genes of the tumor suppressor, retinoblastoma protein, including genes encoding cell cycle regulators such as CDKs, cyclins and CDK inhibitors, are significantly upregulated in malignant mouse tumors in a CDK5-dependent manner. Reducing CDK5 activity in human MTC cells down-regulated these cell cycle regulators suggesting that CDK5 activity is critical for cell cycle progression and MTC proliferation. Finally, the same set of cell cycle proteins was consistently overexpressed in human sporadic MTC but not in hereditary MTC. Together these findings suggest that aberrant CDK5 activity precedes cell cycle initiation and thus may function as a tumor-promoting factor facilitating cell cycle protein expression in MTC. Targeting aberrant CDK5 or its downstream effectors may be a strategy to halt MTC tumorigenesis.

## INTRODUCTION

Neuroendocrine tumors (NETs) are rare cancers originating from hormone-secreting neuroendocrine (NE) cells. These slow-growing neoplasms affect both genders equally and their incidence is rising [1, 2]. NETs are often diagnosed at late metastatic stages due to the absence of specific symptoms and are therefore often fatal. MTC arises from the thyroid parafollicular cells (C-cells), which secrete calcitonin. Most MTC cases (75%) are sporadic and about 40% are caused by somatic mutations in the *RET* proto-oncogene, 15% by mutation in the *RAS* gene, 10% by mutations in other genes and 35% by unknown causes [3-5]. Overall the etiology of sporadic MTC is poorly understood. Hereditary forms of MTC represent about 25% of cases and result from germline mutation in the *RET* proto-oncogene [6]. These genetic forms of MTC are often associated with other types of NE cancers and they are referred to as Multiple Endocrine Neoplasia of Type 2 (MEN 2). Surgical resection of the thyroid is the best treatment currently available for early stage disease but recurrence is common, particularly in sporadic MTC. The prognosis for advanced forms of MTC is poor with a five-year survival rate of 30%. FDA-approved drugs include the tyrosine kinase inhibitors, Vandetanib [7] and Cabozantinib [8], however their efficacy is limited [8, 9]. Therefore a better understanding of the drivers of MTC progression, especially in the absence of *RET* or *RAS* mutations, is needed to develop more effective treatment strategies. Toward this goal, it is paramount to elucidate additional molecular mechanisms underlying MTC and identify new targets for therapy development.

We recently reported that cyclin-dependent kinase 5 (CDK5) was involved in MTC pathogenesis [10, 11]. CDK5 is a serine/threonine kinase that is highly expressed in the brain and regulates neuronal function [12] but its role in cell cycle and cancer has not been well explored. CDK5 is activated by interaction with its cofactor, p35 [13], which can be cleaved by the calcium-dependent protein kinase, calpain, to produce p25. The resulting p25-CDK5 complex engenders aberrant activity with a different range of substrates. CDK5, p35 and p25 are expressed in other tissues besides brain and have been implicated in various forms of neoplasms, including thyroid [10, 11], pancreatic [14, 15], pituitary [16], breast [17], prostate [18, 19], and lung [20] cancers. In particular, CDK5 contributes to MTC by inactivating the tumor suppressor retinoblastoma protein (Rb), which is a ‘gatekeeper’ of the cell cycle [10], thereby suggesting a crucial role for CDK5 in the regulation of the cell cycle.

We have generated a novel conditional MTC mouse model in which overexpression of p25 (p25OE) in mouse thyroid C-cells invokes aberrant CDK5 activity and MTC tumorigenesis [10, 21]. Importantly, in these mice, arrest of p25OE completely halts MTC growth, thereby transforming tumors from a malignant to benign state. Mice harboring arrested tumors exhibit normal survival rates, whereas mice with proliferating MTC die within 30 weeks of transgene induction. A comparison of genes and proteins that are differentially expressed between malignant and benign tumors can help unravel the molecular basis for MTC tumorigenesis. Therefore in this study we investigate further the role of CDK5 in MTC pathogenesis by using an integrated approach including the novel MTC mouse model, human MTC cell lines and patient samples.

## RESULTS

### Differential gene expression analysis of tumors from an inducible medullary thyroid carcinoma mouse model

**Figure 1:**
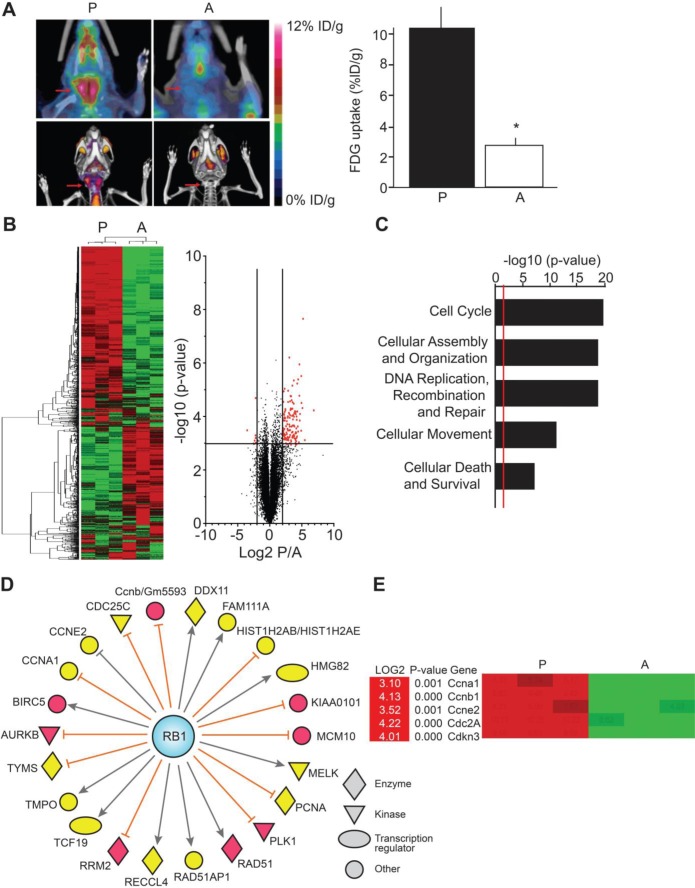
Differential gene expression analysis of the conditional MTC mouse tumors **A**) PET/CT scans show increased thyroid metabolism in mice with proliferating malignant tumors (P) compared to mice with arrested benign tumors (A) (N = 3 for each condition, p = 0.0139, Student's t-test), arrows show the thyroid area. **B**, **C** and **D**) Expression array analyses. **B**) Heat map and volcano plot representations of differentially regulated mRNA in proliferating malignant versus arrested benign mouse tumors. Heat map shows segregation of arrested and proliferating tumors using unsupervised clustering analysis of gene expression data. Green color indicates down-regulated genes and red color, up-regulated genes. The X-axis on volcano plot represents expression of all genes in the microarray, with cutoffs displayed at +/−log2 = 2.00. The Y-axis plots a t-test transform, representing the significance of the gene expression difference for each gene. The line represents the boundary for p-values above –log10 = 3.00. Red points represent genes of interest, which exceeded both indicated cutoffs. **C**) Pathway analysis of up-regulated genes in proliferating tumors. The X-axis represents the p-value associated with each gene category identified from Ingenuity Pathway Analysis (IPA). The higher the value, the less likely that the gene group appears in the proliferating tumors by chance. The red line represents a standard cutoff value of p = 0.01. **D**) IPA analysis showing the relationship between retinoblastoma protein (RB1) and up-regulated downstream molecules that were identified in the gene expression array analysis. Yellow color indicates moderate levels of expression and red color indicates high levels of expression. Relations between Rb and downstream molecules are as follows: orange lines indicate molecules that are inhibited when Rb is in an active, hypophosphorylated state; grey lines indicate that the effect of Rb on the molecule is not predicted. **E**) Examples of cell cycle regulators with quantitation from proliferating vs. arrested tumors. Data are represented as mean +/− SEM.

We have previously described a novel mouse model for MTC in which tumor progression and arrest are induced by overexpressing, and interrupting, green fluorescent protein-tagged p25 (p25-GFP) in thyroid C-cells [10]. Proliferating tumors display abnormally elevated CDK5 activity and are malignant. In contrast, arrested tumors are benign and exhibit much lower levels of CDK5 activity. Consistent with elevated cell proliferation, PET/CT imaging revealed 2.7-fold elevation in metabolic activity for proliferating malignant thyroid tumors compared to arrested benign tumors (Figure 1A). To gain more understanding of the molecular mechanisms underlying p25-CDK5-induced MTC proliferation, we conducted a microarray study of the differential mRNA expression in malignant versus benign tumors. Unsupervised clustering analyses identified 116 genes that were up-regulated, while 7 genes were down-regulated in malignant MTC compared to benign tumors (Figure 1B, Tables S1 and S2). Gene ontology analyses revealed a significant up-regulation of genes involved in cell cycle, cellular assembly and organization, DNA replication, DNA recombination, DNA repair, cellular movement, cellular death and cell survival (Figure 1C).

We previously showed that p25-CDK5-induced tumorigenesis was associated with elevated Rb phosphorylation, which inactivates Rb and leads to increased transcription of E2F target genes [10]. Consistent with these observations, differential gene analysis showed that the expression of E2F target genes such as those encoding Aurora kinase 1, Polo-like kinase 1, Survivin (BIRC5) and the cell cycle regulators CDK1, cyclin-A1 and cyclin-E2 were 3- to 4-fold up-regulated in malignant tumors (Figures 1D-1E, Table S1).

To validate the results of the differential gene expression analyses, *eGFP* was used as an internal control, as *eGFP* mRNA levels increase in proliferating tumors due to tetOp-tTA-mediated p25-GFP transgene overexpression. Consistent with its targeted regulation, the *eGFP* gene exhibited the highest differential expression level (log2 = 6.88, Table S1). Reverse transcription real time PCR (RT-qPCR) analysis confirmed this increased *eGFP* mRNA expression (Figure S1A).

### Gene expression analysis of cell cycle regulators in malignant and benign mouse MTC

While CDK5 is well characterized for its role in the central nervous system, less is known about its role in the cell cycle. We and others have found that CDK5 could regulate the activation state of the tumor suppressor Rb, thereby implicating CDK5 in the regulation of cell cycle progression [10, 22, 23]. Here, the gene expression analysis revealed a significant up-regulation of genes encoding cell cycle proteins, including CDKs, cyclins and endogenous cyclin-dependent kinase inhibitors (CKI), in malignant mouse MTC, suggesting that p25-CDK5-dependent MTC tumorigenesis is associated with alterations in cell cycle regulation.

**Figure 2:**
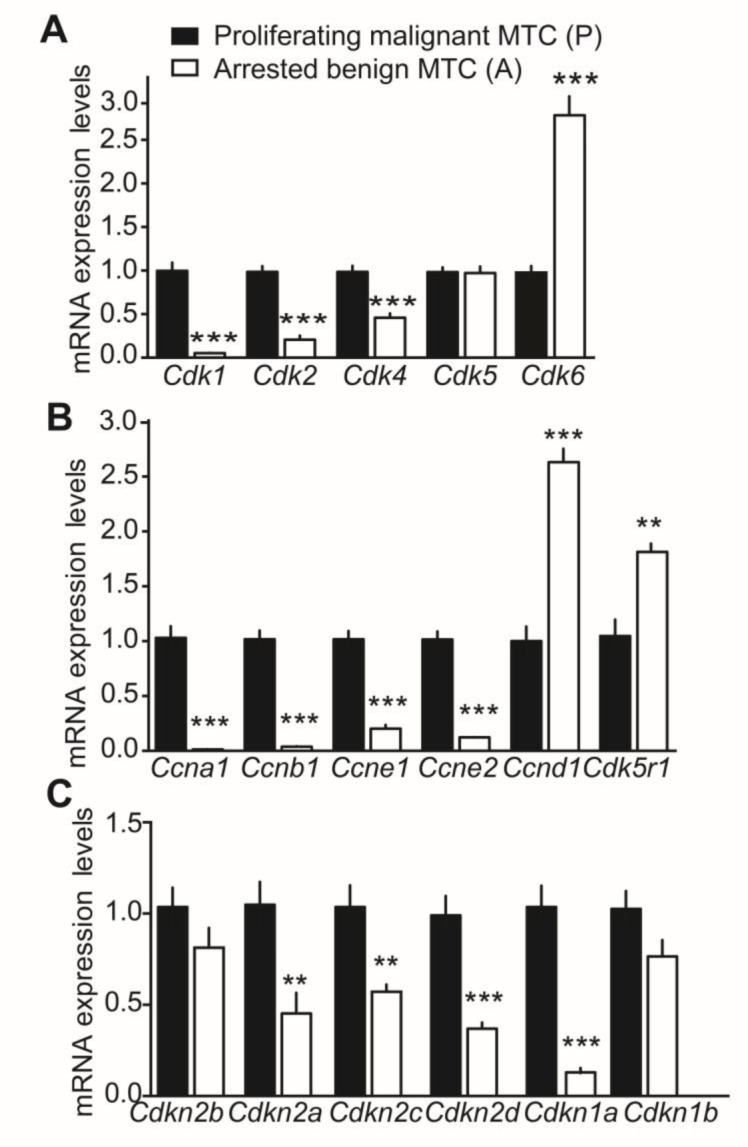
Gene expression analysis of cell cycle regulators in malignant and benign mouse MTC by RT-qPCR Relative mRNA expression of **A**) CDK genes, *Cdk1*, *Cdk2*, *Cdk4, Cdk5* and *Cdk6*; **B**) cyclin genes, *Ccna1*, *Ccnb1*, *Ccne1*, *Ccne*2, *Ccnd1*, and *Cdk5r1* and **C**) CKI genes, *Cdkn2a*, *Cdkn2c*, *Cdkn2d*, Cdkn1a, *Cdkn1b* and *Cdkn2b* in proliferating malignant (P) versus arrested benign NSE/p25-GFP mouse tumors. P-values are p < 0.0001 for *Cdk1*, *Cdk2*, *Cdk4, Cdk6*, p = 0.9838 for CDK5; p < 0.0001 for *Ccna1*, *Ccnb1*, *Ccnd1, Ccne1* and *Ccne*2, p = 0.0057 for *Cdk5r1*; p = 0.1754 for *Cdkn2b*, p = 0.005 for *Cdkn2a*, p = 0.0052 for *Cdkn2c*, p = 0.0003 for *Cdkn2d*, p < 0.0001 for *Cdkn1a* and p = 0.0811 for *Cdkn1b*. Data are represented as mean +/− SEM, N = 6-7 for each condition.

To validate the expression array data and investigate further the role of CDK5 in the cell cycle, we measured the relative expression of genes encoding CDK, cyclins and CKI by RT-qPCR analyses. Elevated *Cdk1*, *Cdk2* and *Cdk4* mRNA levels were detected in proliferating malignant tumors. *Cdk5* mRNA levels remained unchanged despite p25 overexpression. Somewhat unexpectedly, *Cdk6* mRNA expression was increased in arrested benign tumors (Figure 2A). Similarly, cyclin-D1 and p35 gene products, *Ccnd1* and *Cdk5r1*, were up-regulated in benign tumors (Figure 2B). In contrast, the genes encoding cyclin-A1, -B1, -E1 and -E2, *i.e. Ccna1*, *Ccnb1*, *Ccne1* and *Ccne*2, were up-regulated in malignant tumors. Finally, CKI gene expression analysis revealed elevated mRNA expression in malignant tumors for p16^INK4a^, p18^INK4c^, p19^INK4d^ and p21^CIP/WAF1^ encoding genes, *i.e. Cdkn2a*, *Cdkn2c*, *Cdkn2d* and *Cdkn1a*, while p15^INK4b^ and p27^KIP1^ encoding genes, *i.e. Cdkn2b* and *Cdkn1b* mRNA levels were unchanged between conditions (Figure 2C). Thus there is an overall elevation in the expression of several mRNAs encoding CDK, cyclins and CKI in proliferating malignant tumors compared to arrested benign MTC, thereby suggesting a role for CDK5 activity in the cell cycle.

### Protein expression analysis of cell cycle regulators in malignant and benign mouse MTC

**Figure 3:**
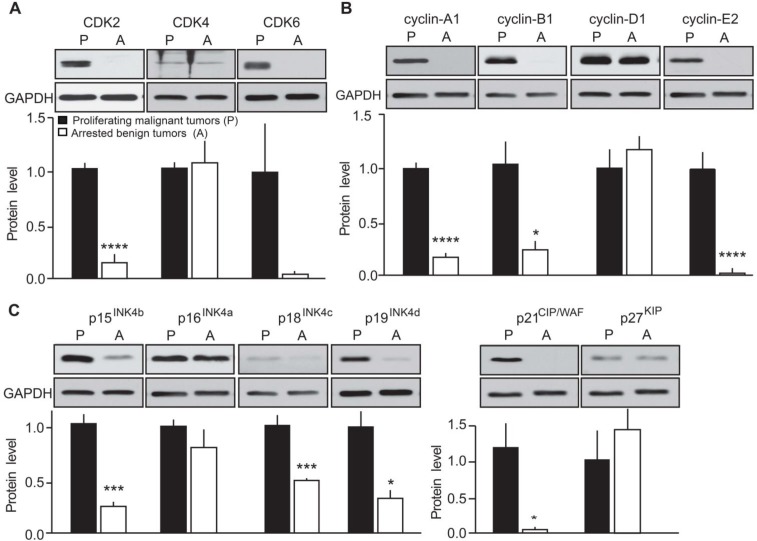
Evaluation of the effects of p25-GFP overexpression on cell cycle protein expression in malignant versus benign mouse MTC Immunoblots of lysates from proliferating malignant (P) and arrested benign (A) mouse MTC for **A**) CDK2, CDK4 and CDK6; **B**) cyclins A1, B1, D1, E2 and **C**) p15^INK4b^, p16^INK4a^, p18^INK4c^, p19^INK4d^, p21^CIP/WAF1^ and p27^KIP^. P-values are p < 0.0001 for CDK2, p = 0.6974 for CDK4, p = 0.0633 for CDK6; p < 0.0001 for cyclin-A1, p = 0.0194 for cyclin-B1, p = 0.3416 for cyclin-D1, p < 0.0001 for cyclin-E2; p = 0.0002 for p15^INK4b^, p = 0.291 for p16^INK4a^, p = 0.0006 for p18^INK4c^, p = 0.0054 for p19^INK4d^, p = 0.02 for p21^CIP/WAF^ and p = 0.67 for p27^KIP1^. Data are represented as mean +/− SEM, N = 6 for each condition.

To further confirm the effects of p25-CDK5 up-regulation on expression of cell cycle regulators, we evaluated protein expression levels in proliferating malignant versus arrested benign mouse MTC (Figure 3). Quantification of immunoblots revealed CDK2 protein levels were elevated in proliferating tumors as previously observed [10]. However, CDK4 expression was unchanged between conditions (Figure 3A). CDK6 levels were more variable from sample to sample and thus no significant change in CDK6 was detected between malignant and benign MTC (Figure 3A). Consistent with mRNA findings, protein levels of cyclin-A1 [10], cyclin-B1 and cyclin-E2 were all up-regulated in malignant MTC (Figure 3B). Cyclin-D1 protein expression was also variable from sample to sample and overall its expression was not significantly altered (Figure 3B). As observed at the mRNA levels, CKI protein expression was predominantly up-regulated in malignant mouse tumors. In particular, the expression of p15^INK4b^, p18^INK4c^, p19^INK4d^ and p21^CIP/WAF1^ was elevated in proliferating tumors compared to those in the arrested state (Figure 3C). In contrast, the expression of p16^INK4a^ and p27^KIP1^ was not significantly changed between conditions. Overall, these results are consistent with the observations from the differential gene expression analysis.

### Evaluation of the relationship between CDK5 activity and cell cycle protein expression levels

The gene and cell cycle protein expression analyses described above indicate a possible relationship between CDK5 activity and the regulation of cell cycle protein expression in proliferating versus arrested mouse MTC tumors. To directly assess the effect of CDK5 activity on the expression of cell cycle regulators in human MTC, we compared cell cycle protein expression in a human sporadic, non-RET mutated MTC cell line, MTC-SK [24], which was transfected with either a construct encoding kinase-dead CDK5 (Kd-CDK5) or with a control plasmid. We previously demonstrated that overexpressing kinase-dead CDK5 in MTC-SK cells abrogates CDK5 activity and stops cell proliferation [10].

**Figure 4:**
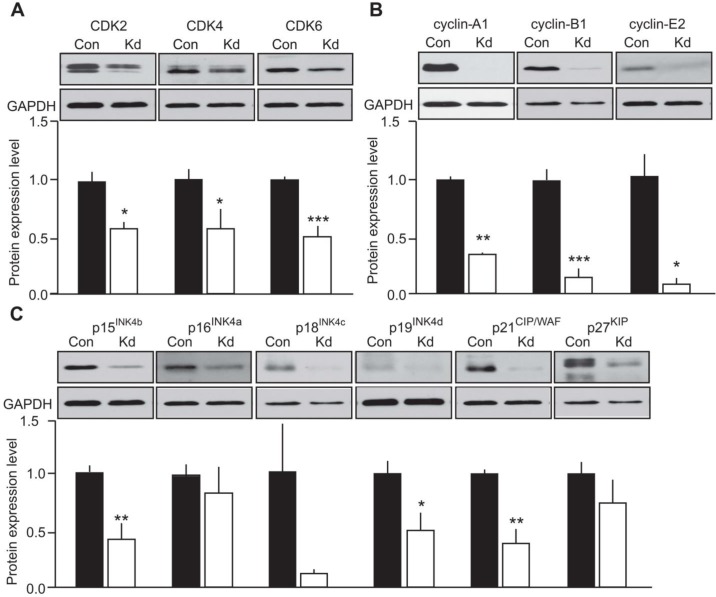
Evaluation of the effect of CDK5 activity on cell cycle protein expression in human MTC cells Immunoblots of lysates from MTC-SK cells transfected with either a control plasmid (Con) or a kinase-dead (Kd) CDK5 encoding plasmid for **A**) CDK2, CDK4 and CDK6; **B**) cyclin-A1,-B1,-E2 and **C**) p15^INK4b^, p16^INK4a^, p18^INK4c^, p19^INK4d^, p21^CIP/WAF1^ and p27^KIP^. P-values are p = 0.0244 for CDK2, p = 0.038 for CDK4, p = 0.009 for CDK6; p < 0.0001 for cyclin-A1, p = 0.0005 for cyclin-B1, p = 0.0116 for cyclin-E2; p = 0.0044 for p15^INK4b^, p = 0.4687 for p16^INK4a^, p = 0.1032 for p18^INK4c^, p = 0.0195 for p19^INK4d^, p = 0.0022 for p21^CIP/WAF^ and p = 0.2674 for p27^KIP1^. Data are represented as mean +/− SEM, N = 4-6 for each condition.

As a prerequisite observation, most of the CDKs, cyclins and CKI that were detected in the mouse tumors were also expressed in MTC-SK cells transfected with a control plasmid (Figure 4). The only exception was cyclin-D1 which was not detected in MTC-SK cells and was unaffected by CDK5 activity in growing versus arrested mouse tumors. Interestingly, the expression of CDK2, CDK4 and CDK6 was diminished following Kd-CDK5 overexpression (Figure 4A). Moreover, the protein levels of cyclin-A1, cyclin-B1 and cyclin-E2 were all reduced in Kd-CDK5-transfected cells (Figure 4B). Finally, the expression of p15^INK4b^, p19^INK4d^ and p21^CIP/WAF1^ was decreased in transfected MTC-SK cells, whereas the protein levels of p16^INK4a^, p18^INK4c^ and p27^KIP1^ were not significantly changed (Figure 4C). These observations are consistent with the analysis of cell cycle protein expression in the mouse MTC. Together these results suggest that CDK5 activity regulates the expression of CDK2, cyclin-A1, cyclin-B1, cyclin-E2, p15^INK4b^, p19^INK4d^ and p21^CIP/WAF1^ but not p16^INK4a^ and p27^KIP1^ (Table 1). It is not clear whether CDK5 modulates the expression of CDK4, CDK6 and p18^INK4c^. Overall, these findings are in agreement with CDK5 regulating the expression of Rb-E2F target genes.

### Analysis of cell cycle protein expression in human MTC tissue

**Figure 5:**
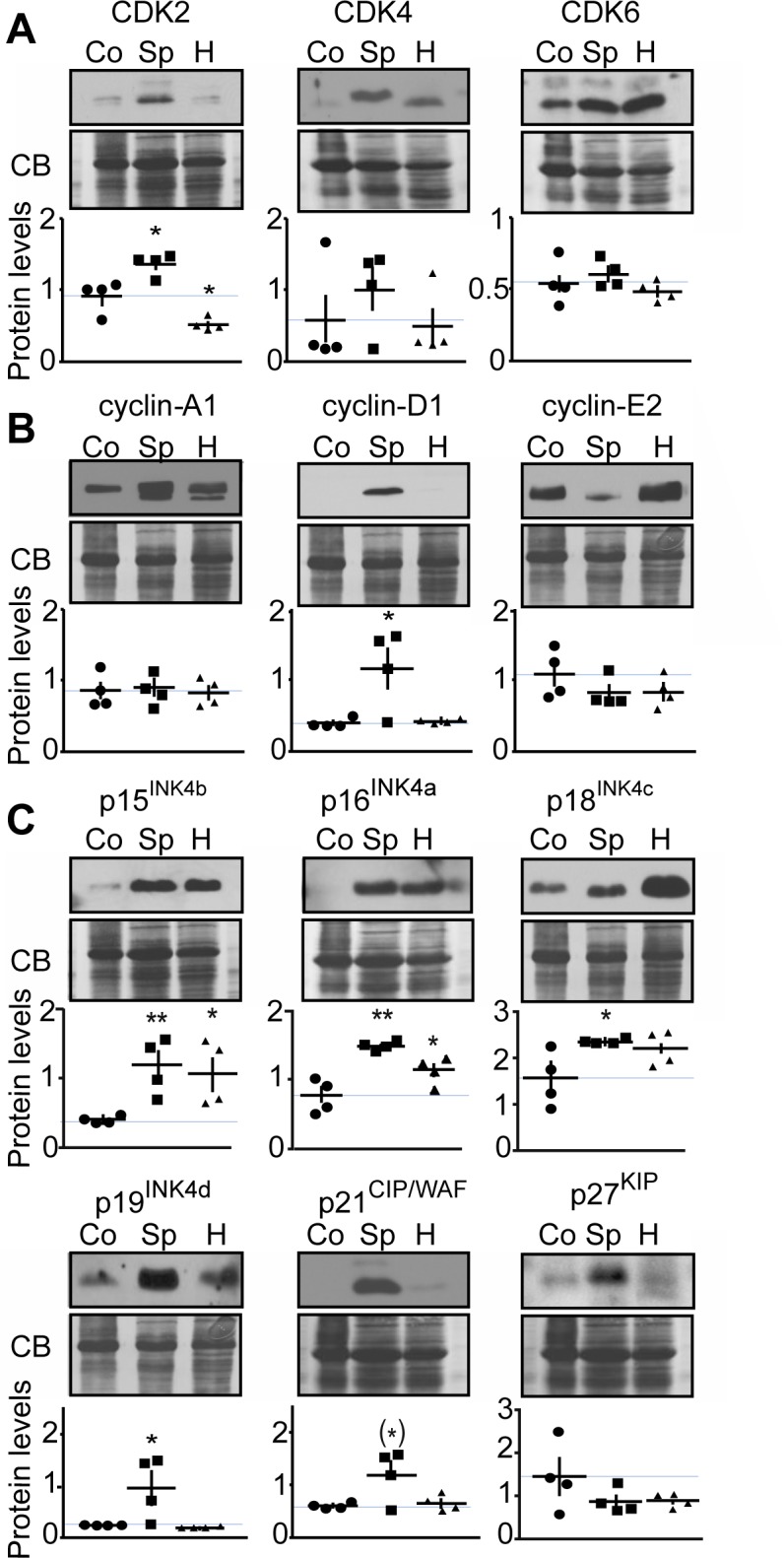
Analysis of cell cycle protein expression in human MTC samples Representative immunoblots of lysates from control thyroid tissue (Co), sporadic (Sp) and hereditary (H) human MTC tumors with antibodies as indicated are shown with quantification. Protein levels were normalized to Coomassie blue (CB) signal. Immunoblots were probed with antibodies to **A**) CDK2, CDK4 and CDK6; **B**) cyclins-A1, -D1, -E2; **C**) in p15^INK4b^, p16^INK4a^, p18^INK4c^, p19^INK4d^, p21^CIP/WAF1^ and p27^KIP^. P-values were for CDK2, Sp, p = 0.0168 and H, p = 0.0140; for CDK4, Sp, p = 0.4072 and H, p = 0.8576; for CDK6, Sp, p = 0.5484 and H, p = 0.4916; for cyclin-A1, Sp, p = 0.9923 and H, p = 0.7017; for cyclin-D1, Sp, p = 0.0307 and H, p = 0.6883; for cyclin-E2, Sp, p = 0.2645 and H, p = 0.3070; for p15^INK4b^, Sp, p = 0.0088 and H, p = 0.0310; for p16^INK4a^, Sp, p = 0.0014 and H, p = 0.0496; for p18^INK4c^, Sp, p = 0.0309 and H, p = 0.1054; for p19^INK4d^, Sp, p = 0.0484 and H, p = 0.3560; for p21^CIP/WAF^, Sp, p = 0.0554 and H, p = 0.6807; for p27^KIP1^, Sp, p = 0.2149 and H, p = 0.2131. Data are represented as mean +/− SEM, N = 4 for each condition.

While hereditary MTC is mainly caused by RET proto-oncogene mutations and consequent deregulation of the RET signaling pathway, the molecular basis for sporadic MTC is not well understood. Some sporadic MTC cases harbor mutations in the *RET* or *Ras* genes, but others do not. We previously reported that CDK5 was involved in MTC tumorigenesis, and found that high levels of CDK5 and its activators, p35 and p25, occur predominantly in the sporadic compared to the hereditary form of the disease [10]. Having established that CDK5 activity correlates with elevated cell cycle protein expression levels in proliferating mouse MTC and in a human sporadic MTC cell line, we compared protein levels of CDKs, cyclins and CKI in sporadic and hereditary MTC patient tissues (Figure 5). Sporadic specimen did not exhibit RET mutations (see method section). We found that CDK2 levels were elevated in sporadic cases, but decreased in hereditary forms of MTC. In contrast, CDK4 and CDK6 showed no significant changes in any of the groups analyzed (Figure 5A). Cyclin-D1 levels were increased in sporadic MTC, while cyclin-A1 and cyclin-E2 were unchanged in MTC compared to control thyroid samples (Figure 5B). Cyclin-B1 could not be detected in control thyroid or MTC specimens. p15^INK4b^ and p16^INK4a^ expression was increased in both, sporadic and hereditary MTC. The levels of p18^INK4c^, p19^INK4d^ and p21^CIP/WAF1^ were elevated in sporadic but not hereditary MTC. Finally p27^KIP1^ expression was unchanged in MTC samples compared to control samples. Proliferating Cell Nuclear Antigen was expressed at the same level in sporadic and hereditary tumors, thereby confirming that the observed changes were not just a measure of growth fraction (Figure S1B). The changes in cell cycle regulator expression that were observed in sporadic MTC tissues are consistent with those observed in the human MTC cell line and in mouse tumors (Table 1). The results suggest that the MTC mouse model may more accurately model the molecular mechanisms underlying sporadic MTC than hereditary forms. Thus CDK5 may play a more important role in sporadic than familial MTC tumorigenesis.

**Table 1:** A Table summarizing cell cycle protein expression in mouse MTC tumors, human MTC-SK cells and human MTC tissues

	Mouse model		Human cell line	Patient tissues		
	Arrested proliferating MTC	vs	kd-Cdk5-transfected cells vs control cells	Sporadic MTC control thyroid	vs	Hereditary MTC vs control thyroid
Cdk2	↓		↓	↑		↓
Cdk4	-		↓	-		-
Cdk6	-		↓	-		-
cyclin A1	↓		↓	-		-
cyclin B1	↓		↓	n.d.		n.d.
cyclin D1	-		n.d.	↑		-
cyclin E2	↓		↓	-		-
p15^INK4b^	↓		↓	↑		↑
p16^INK4a^	-		-	↑		↑
p18^INK4c^	↓		-	↑		-
p19^INK4d^	↓		↓	↑		-
p21^CIP/WAF1^	↓		↓	(↑)		-
p27^KIP1^	-		-	-		-

To determine the role of CDK5 activity in the expression of cell cycle regulators, we assessed the changes in protein level of cyclins, CDKs, and CKI in mouse tumors and in MTC-SK cells following blockade of CDK5 activity. We have indeed previously demonstrated that CDK5 activity is reduced in arrested tumors by stopping p25-GFP transgene expression and in MTC-SK cells by overexpressing a kinase-dead CDK5 construct [10]. The changes in expression of cell cycle regulators in human MTC tumors compared to normal thyroid tissues are also reported.

**Figure 6:**
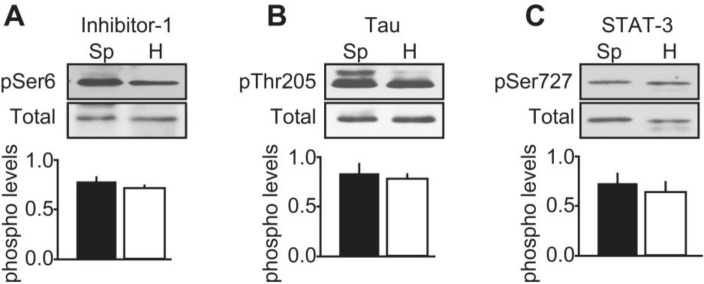
Characterization of CDK5 activity in human MTC tissues Representative immunoblots of lysates from sporadic (Sp) and hereditary (H) MTC tumors with antibodies to **A**) inhibitor-1 and phospho-Ser6 Inhibitor-1; **B**) Tau and phospho-Thr205 Tau and C) STAT-3 and phospho-Ser727 STAT-3. Phosphorylated levels were normalized to total levels. P-values were p = 0.2061 for inhibitor-1; p = 0.8545 for Tau and p = 0.6740 for STAT-3. Data are represented as mean +/− SEM, N = 4 for each condition.

To characterize further CDK5 activity in human tumors, we compared the phosphorylation state of the known CDK5 substrates, inhibitor-1 (Ser-6) [25], Tau (Thr205) [26] and STAT-3 (Ser727) [27] between sporadic and hereditary tumors (Figure 6). We found these substrates were phosphorylated at their respective CDK5 sites, equally in both forms of MTC. Thus CDK5 activity is not uniformly elevated in response to p25 overexpression, and as p35 expression remains normal in both forms of MTC [10], the phosphorylation state of physiological CDK5 substrates may be unaffected.

Together the analysis of human tumors, cell line and mouse MTC suggests that up-regulation of cell cycle protein expression via a CDK5-mediated mechanism may contribute to sporadic MTC pathogenesis. The role of CDK5 in hereditary MTC tumorigenesis such as those arising from familial mutations in RET is less clear. These findings also suggest that cell cycle proteins may serve as useful biomarkers for sporadic forms of neuroendocrine thyroid cancer.

## DISCUSSION

MTC is a devastating disease for which new treatments are urgently needed. Having generated a novel, inducible animal model for MTC, we set to elucidate the molecular mechanisms underlying this cancer. Array-based transcriptome analysis comparing proliferating malignant and arrested benign mouse MTC revealed deregulation of cell cycle regulator expression (Figure 1). A focused examination uncovered a CDK5-dependent increase in expression of genes encoding cell cycle regulators, including CDK2, cyclin-D1, p15^INK4b^, p16^INK4a^, p18^INK4c^, p19^INK4d^ and p21^CIP/WAF1^. RT-qPCR and protein expression analyses confirmed their up-regulation at mRNA and protein level in p25-overexpressing malignant mouse MTC. Furthermore, the same set of cell cycle proteins are down-regulated in human MTC cells lacking CDK5 activity, suggesting aberrant CDK5 activity is necessary and sufficient to drive expression of these markers. Importantly, there was no difference in levels of CDK5 itself, thereby underlining that it is the aberrant activity of the kinase that is critical to these malignancy-associated effects rather than its expression level. Analysis of human MTC tissues from sporadic and hereditary cases showed that the cell cycle proteins, CDK2, cyclin-D1, p18^INK4c^, p19^INK4d^ and p21^CIP/WAF1^ were consistently overexpressed in human sporadic, but not hereditary, forms of MTC (Table 1). Although we could not demonstrate that CDK5 activity is higher in sporadic than in hereditary tumors, the integrated analysis of mouse tumors, the human cell line and patient samples suggest that CDK5 activity may be responsible for the increase in cell cycle protein expression that is detected in sporadic, non-RET MTC tissues. Our observations do not allow excluding a role for CDK5 in RET-dependent hereditary MTC. Cdk5 and its activators are indeed expressed in some hereditary tumors [10] and CDK5 has also been suggested to contribute to the proliferation of TT cells, which are derived from a hereditary form of MTC (RET mutation on codon 634) [28]. It will be important in the future to determine whether CDK5 is involved in RET-mediated MTC pathogenesis and how.

Here we find that all the members of the INK4 family of CKI, which are four ankyrin-repeat domains proteins inhibiting only cyclin-D-CDK4/6 function [29], are up-regulated in MTC patients. In particular p15^INK4b^ and p16^INK4a^ expression is increased in non-RET sporadic and RET-mutated, hereditary MTCs while p18^INK4c^ and p19^INK4d^ levels are elevated in non-RET sporadic MTC only. Interestingly p18^INK4c^ and p19^INK4d^ have potential CDK5 phosphorylation sites whereas p15^INK4b^ and p16^INK4a^ do not [30], thereby supporting our hypothesis that CDK5 may be more important for sporadic than hereditary MTC. The INK4 family members have previously been related to MTC. Base pair change mutations in exon 2 of the p15^INK4b^-encoding gene were found in some MTC cases [31]. Somatic mutations in exon 3 of the p18^INK4c^-encoding gene disrupting p18^INK4c^ interaction with CDK4 or CDK6 were also detected in MTC and pheochromocytoma patients [32]. In addition, mutations in other exons of the p18^INK4c^-encoding gene or within p19^INK4d^-encoding gene have been reported in MTC patients [33]. Mutant mice carrying deletions of p18^INK4c^-encoding gene as well as the p27^KIP1^-encoding gene develop multiple endocrine tumors including MTC and pheochromocytoma [34, 35]. Finally, deletion of the p18^INK4c^-encoding gene and/or the p27^KIP1^-encoding gene amplifies the tumorigenic effect of RET mutations causing hereditary forms of MTC as studied in mouse models and cell culture [35, 36]. Together with our findings, these observations lead us to propose that inactivation or loss of p18^INK4c^ and p19^INK4d^ might be a feature of RET-dependent hereditary MTC, while overexpression of these proteins might characterize non-RET sporadic MTC. Analysis of large cohorts of RET and non-RET MTC specimen will be necessary to validate this hypothesis.

In this study, we present compelling evidence that CDK5 functions in the regulatory mechanisms that govern cell cycle progression and contribute to MTC tumorigenesis. The fact that a given set of cell cycle proteins display the same expression profile in three different MTC models, namely mouse model tissues, human sporadic MTC cell lines, and MTC patient samples, serves to underscore the relevance of our observations to mechanisms underlying this disease.

The cell cycle is initiated after the restriction point, or step of no-return, at which cells irreversibly commit to DNA replication. Bypassing the restriction point requires phosphorylation of Rb by CDKs, namely cyclin-D1-CDK4/6. Thereafter, cell cycle progression is reliant on coordinated action of cyclin-CDK complexes at defined phases throughout the cell cycle. Based on our current and previous studies, we propose that the aberrant activity of CDK5 may facilitate bypass of the restriction point through initial deactivation of Rb, via phosphorylation, which in turn promotes E2F target gene expression. In support of this notion, we show here that E2F target genes, including CDK2, p15^INK4b^, p19^INK4d^ and p21^CIP/WAF1^ [37, 38] are up-regulated in p25-overexpressing malignant mouse MTCs, while the expression of these proteins is reduced following inhibition of CDK5 activity.

In summary, this study suggests that aberrant CDK5 activity promotes sporadic forms of MTC by modulating the expression of cell cycle proteins and points to these as possible biomarkers or targets for the development of new therapies.

## MATERIALS AND METHODS

### Human samples

Medullary thyroid cancer specimens and control thyroid samples (goiters) were obtained through a human subject institutional-review-board-approved protocol UT Southwestern IRB 052004-044, ‘‘Molecular Analysis of Endocrine Tumors’’.

### Sequencing of human MTC tumors and of the MTC-SK cell line

MTC tumors and MTC-SK cells were examined for ‘hot spot’ somatic RET mutations (exons 10, 11, 13, 14, 15 and 16) by direct sequencing of DNA extracted from tumor specimens using a QIAamp DNA FFPE Tissue Kit (QIAgen) or extracted from cultured MTC-SK cells using DNAeasy Blood and Tissue Kit (QIAgen) according to the manufacturer's instructions. Sequencing was performed as previously described with some modification of primer sequences and conditions [39]. See supplemental methods for detailed information regarding primer sequences and reaction conditions. Sporadic MTC tumors (n = 4) had no mutations in RET exons. Hereditary MTC samples exhibited either a RET C634R mutation (n = 2) or a RET C618S mutation (n = 1), or RET L790F (n = 1). Because of the low number of available samples, tumors were classified as either sporadic (non-RET) or hereditary (RET-mutated). No RET mutations were detected in MTC-SK cells.

### Animals

The NSE/p25-GFP line is a bitransgenic mouse model of MTC based on a tetracycline transactivator system and has been described in detail [10, 21]. Briefly the neuron specific enolase (NSE) promotor controls expression of a tetracycline transactivator that is inhibited by dietary doxycycline (i.e., Dox off). Thus removing doxycycline (100 mg/L) from drinking water induces p25-GFP expression and MTC development. Subsequent re-addition of doxycycline stops p25-GFP overexpression and arrests tumor growth.

### Tumor collection and lysate preparation

Proliferating tumors were obtained by inducing p25-GFP expression in NSE/p25-GFP mice for 16 weeks. For arrested tumors, NSE/p25-GFP mice were deprived from doxycycline for 16 weeks and exposed to doxycycline for 4 weeks. Tumors were collected and lysates were prepared as described in [40].

### Positron Emission Tomography (PET)/Computed Tomography (CT) imaging

Mouse PET/CT imaging was performed using a Siemens Inveon PET/CT Multi Modality system (Siemens Medical Solutions, Knoxville, TN) with effective spatial resolution of 1.4 mm at the center of field of view (FOV). All animals were fasted for 12 h prior to PET imaging. Each mouse received 140 μCi of 2-deoxy-2-(18F)fluoro-D-glucose (FDG) in 150 μL in saline intravenously via tail vain injection. The mice were placed on a heat pad before and during image acquisition. PET images were acquired 1h post-injection (p.i.), for 15 min, with animals under 2.5% isoflurane. PET images were reconstructed into a single frame using the 3D Ordered Subsets Expectation Maximization (OSEM3D/MAP) algorithm. CT images were acquired immediately after PET with the FOV centered at the shoulder of the mouse. CT projections (360 steps/rotation) were acquired with a power of 80 kVp, current of 500 μA, exposure time of 145 ms, binning of 4, and effective pixel size of 102 μm. The CT reconstruction protocol used a downsample factor of 2, was set to interpolate bilinearly, and used a Shepp-Logan filter. PET and CT images were co-registered in Inveon Acquisition Workplace (Siemens Medical Solutions) for analysis. Regions of interest (ROI) were drawn manually, encompassing the thyroid in all planes containing the organ. The target activity was calculated as percentage injected dose per gram.

### Microarray and pathway analyses

For microarray analysis, total RNA from arrested and proliferating tumors was isolated using RNeasy kit (QIAGEN, Hilden, Germany). Gene expression profiling on each sample was performed using Illumina Mouse WG-6 V3 BeadArrays (San Diego, CA, USA). Bead-level data were obtained and pre-processed using the R package “mbcb” for background correction and probe summarization. Pre-processed data were then quartile-normalized and log-transformed. Class comparison and unsupervised hierarchical clustering was performed using in-house MATRIX 1.48 (Girard, L. Manuscript in preparation). The volcano plot was developed by transforming p-values obtained in the *t*-test and plotting the transformed p-value versus the log2 expression value obtained following microarray data processing in MATRIX 1.48. The networks and functional analyses were generated through the use of QIAGEN's Ingenuity Pathway Analysis (IPA® QI Redwood City, www.qiagen.com/ingenuity).

### Reverse transcription real time PCR (RT-qPCR)

Tissues of proliferating malignant (n = 7) and arrested benign tumors (n = 6) were collected as described above and homogenized in Trizol reagent (Life Technologies). RNA was extracted using QIAGEN RNeasy Kit according to the supplier's protocol. Purified RNA was treated with RNase-free recombinant DNase I (Roche Diagnostics GmbH). Reverse transcription was performed using iScript select cDNA Synthesis Kit with provided random primers according to manufacturer's instructions (Biorad Laboratories, Hercules, CA, USA). The SYBR-green based DyNAmo Flash SYBR Green qPCR Kit (Thermo scientific, Waltham, MA, USA) containing ROX as an internal reference dye was used for amplification. Real-time PCR reactions were run on an AB 7500 Real-time PCR system (Applied Biosystems, Foster City, CA, USA) and the specificity of each reaction was controlled by melt curve analysis. Primers were purchased from Integrated DNA Technologies (Coralville, IA, USA) and designed as intron-spanning pairs when possible (Table S4). Relative expression levels were calculated according to the 2-ΔΔCt method [41]. Expression levels were normalized to Beta-2 microglobulin as endogenous control and calibrated to average expression level of proliferating tumors for each gene. Unpaired t-tests were used to compare changes in expression for each gene.

### Cell culture, transfections and cell lysate preparation

MTC-SK cells were used for cell culture experiments. Those cells were derived from sporadic (non MEN) tumors and do not harbor RET mutations. MTC-SK cells were maintained in culture media containing Ham's F12 (Lonza Group Ltd, Basel, Switzerland), Medium 199 (Sigma, St. Louis, MO, USA) 1:1 v/v and 10% FBS as described in [24]. For transfections, cells were plated at a density of 2.5x10^5^ cells/ml and transfected with either 1 μg pCMV-Kd-CDK5 [34] or 1 μg pCMV-EGFP (Clontech Laboratories, Inc., Mountain View, CA, USA) using X-tremeGene HP DNA transfection reagent (Roche Diagnostics GmbH, Mannheim, Germany). Cells were harvested 24 h post-transfection and lysate was prepared as previously described in [42].

### Immunoblotting

Immunoblotting was conducted as previously described [43]. Membranes were probed with antibodies to p16^INK4a^ (PA5-20379, Pierce, Rockford, IL; USA), p15^INK4b^ (4822, Cell Signaling Technology (CST), Danvers, MA, USA), p18^INK4c^ (39-3400, Life Technologies, Carlsbad, CA, USA), p19^INK4d^ (PA5-26413, Pierce), p21^CIP/WAF^ (2946, CST), p27^KIP^ (2552, CST), CDK2 (sc-163, Santa Cruz Biotechnology (scbt), Santa Cruz, CA, USA), CDK4 (ab7955, Abcam, Cambridge, MA, USA), CDK6 (ABC275, Millipore, Temecula, CA, USA), Cyclin-A1 (sc-596, scbt), Cyclin-B1 (4135, CST), Cyclin D1 (ab134175, Abcam), Cyclin-E2 (4132, CST), GAPDH (G8795, Sigma), Tubulin (T5168, Sigma), STAT-3 (9132, CST) and pS727-STAT-3 (9134, CST), Tau (DAKO), pThr205-Tau (T6694, Sigma). Antibodies to Inhibitor-1 and pS6-inhibitor-1 were generated and characterized in-house [25]. The same membrane was probed for several proteins of different molecular weights after stripping in buffer containing 61 mM Tris-Base, 2%SDS and 7% β-mercaptoethanol at 50°C for 30 min. Immunoblots were quantified using Quantity One (BioRad). Samples were normalized to GAPDH (Figures 3 and 4) or to total protein levels as determined by Coomassie blue (CB) staining (Figure 5).

### Statistical analysis

Results are presented as mean values and error bars represent ± SEM. Statistical analyses were conducted using two-tailed Student's *t*-test in GraphPad Prism 6.0 (GraphPad Software, Inc., La Jolla, CA, USA) and p-values < 0.05 were considered as statistically significant.

## SUPPLEMENTARY MATERIAL, FIGURES, TABLES



## References

[1] Chen H, Sippel RS, O'Dorisio MS, Vinik AI, Lloyd RV, Pacak K (2010). The North American Neuroendocrine Tumor Society consensus guideline for the diagnosis and management of neuroendocrine tumors: pheochromocytoma, paraganglioma, and medullary thyroid cancer. Pancreas.

[2] Yao JC, Hassan M, Phan A, Dagohoy C, Leary C, Mares JE, Abdalla EK, Fleming JB, Vauthey JN, Rashid A, Evans DB (2008). One hundred years after “carcinoid”: epidemiology of and prognostic factors for neuroendocrine tumors in 35,825 cases in the United States. Journal of clinical oncology : official journal of the American Society of Clinical Oncology.

[3] Hofstra RM, Landsvater RM, Ceccherini I, Stulp RP, Stelwagen T, Luo Y, Pasini B, Hoppener JW, van Amstel HK, Romeo G (1994). A mutation in the RET proto-oncogene associated with multiple endocrine neoplasia type 2B and sporadic medullary thyroid carcinoma. Nature.

[4] Moura MM, Cavaco BM, Pinto AE, Leite V (2011). High prevalence of RAS mutations in RET-negative sporadic medullary thyroid carcinomas. The Journal of clinical endocrinology and metabolism.

[5] Agrawal N, Jiao Y, Sausen M, Leary R, Bettegowda C, Roberts NJ, Bhan S, Ho AS, Khan Z, Bishop J, Westra WH, Wood LD, Hruban RH, Tufano RP, Robinson B, Dralle H (2013). Exomic sequencing of medullary thyroid cancer reveals dominant and mutually exclusive oncogenic mutations in RET and RAS. The Journal of clinical endocrinology and metabolism.

[6] Mulligan LM, Kwok JB, Healey CS, Elsdon MJ, Eng C, Gardner E, Love DR, Mole SE, Moore JK, Papi L (1993). Germ-line mutations of the RET proto-oncogene in multiple endocrine neoplasia type 2A. Nature.

[7] Wells SA, Robinson BG, Gagel RF, Dralle H, Fagin JA, Santoro M, Baudin E, Elisei R, Jarzab B, Vasselli JR, Read J, Langmuir P, Ryan AJ, Schlumberger MJ (2012). Vandetanib in patients with locally advanced or metastatic medullary thyroid cancer: a randomized, double-blind phase III trial. Journal of clinical oncology : official journal of the American Society of Clinical Oncology.

[8] Elisei R, Schlumberger MJ, Muller SP, Schoffski P, Brose MS, Shah MH, Licitra L, Jarzab B, Medvedev V, Kreissl MC, Niederle B, Cohen EE, Wirth LJ, Ali H, Hessel C, Yaron Y (2013). Cabozantinib in progressive medullary thyroid cancer. Journal of clinical oncology : official journal of the American Society of Clinical Oncology.

[9] Sherman SI (2013). Lessons learned and questions unanswered from use of multitargeted kinase inhibitors in medullary thyroid cancer. Oral oncology.

[10] Pozo K, Castro-Rivera E, Tan C, Plattner F, Schwach G, Siegl V, Meyer D, Guo A, Gundara J, Mettlach G, Richer E, Guevara JA, Ning L, Gupta A, Hao G, Tsai LH (2013). The role of Cdk5 in neuroendocrine thyroid cancer. Cancer cell.

[11] Pozo K, Nwariaku FE, Bibb JA (2014). Breaking Bad: how does a neuronal protein cause neuroendocrine cancer?. Oncotarget.

[12] Angelo M, Plattner F, Giese KP (2006). Cyclin-dependent kinase 5 in synaptic plasticity, learning and memory. Journal of neurochemistry.

[13] Hisanaga S, Saito T (2003). The regulation of cyclin-dependent kinase 5 activity through the metabolism of p35 or p39 Cdk5 activator. Neuro-Signals.

[14] Eggers JP, Grandgenett PM, Collisson EC, Lewallen ME, Tremayne J, Singh PK, Swanson BJ, Andersen JM, Caffrey TC, High RR, Ouellette M, Hollingsworth MA (2011). Cyclin-dependent kinase 5 is amplified and overexpressed in pancreatic cancer and activated by mutant K-Ras. Clinical cancer research : an official journal of the American Association for Cancer Research.

[15] Feldmann G, Mishra A, Hong SM, Bisht S, Strock CJ, Ball DW, Goggins M, Maitra A, Nelkin BD (2010). Inhibiting the cyclin-dependent kinase CDK5 blocks pancreatic cancer formation and progression through the suppression of Ras-Ral signaling. Cancer research.

[16] Xie W, Wang H, He Y, Li D, Gong L, Zhang Y (2014). CDK5 and its activator P35 in normal pituitary and in pituitary adenomas: relationship to VEGF expression. International journal of biological sciences.

[17] Liang Q, Li L, Zhang J, Lei Y, Wang L, Liu DX, Feng J, Hou P, Yao R, Zhang Y, Huang B, Lu J (2013). CDK5 is essential for TGF-beta1-induced epithelial-mesenchymal transition and breast cancer progression. Scientific reports.

[18] Strock CJ, Park JI, Nakakura EK, Bova GS, Isaacs JT, Ball DW, Nelkin BD (2006). Cyclin-dependent kinase 5 activity controls cell motility and metastatic potential of prostate cancer cells. Cancer research.

[19] Hsu FN, Chen MC, Chiang MC, Lin E, Lee YT, Huang PH, Lee GS, Lin H (2011). Regulation of androgen receptor and prostate cancer growth by cyclin-dependent kinase 5. The Journal of biological chemistry.

[20] Demelash A, Rudrabhatla P, Pant HC, Wang X, Amin ND, McWhite CD, Naizhen X, Linnoila RI (2012). Achaete-scute homologue-1 (ASH1) stimulates migration of lung cancer cells through Cdk5/p35 pathway. Molecular biology of the cell.

[21] Meyer DA, Richer E, Benkovic SA, Hayashi K, Kansy JW, Hale CF, Moy LY, Kim Y, O'Callaghan JP, Tsai LH, Greengard P, Nairn AC, Cowan CW, Miller DB, Antich P, Bibb JA (2008). Striatal dysregulation of Cdk5 alters locomotor responses to cocaine, motor learning, and dendritic morphology. Proceedings of the National Academy of Sciences of the United States of America.

[22] Hamdane M, Bretteville A, Sambo AV, Schindowski K, Begard S, Delacourte A, Bertrand P, Buee L (2005). p25/Cdk5-mediated retinoblastoma phosphorylation is an early event in neuronal cell death. Journal of cell science.

[23] Futatsugi A, Utreras E, Rudrabhatla P, Jaffe H, Pant HC, Kulkarni AB (2012). Cyclin-dependent kinase 5 regulates E2F transcription factor through phosphorylation of Rb protein in neurons. Cell cycle (Georgetown, Tex).

[24] Pfragner R, Hofler H, Behmel A, Ingolic E, Walser V (1990). Establishment and characterization of continuous cell line MTC-SK derived from a human medullary thyroid carcinoma. Cancer research.

[25] Nguyen C, Nishi A, Kansy JW, Fernandez J, Hayashi K, Gillardon F, Hemmings HC, Nairn AC, Bibb JA (2007). Regulation of protein phosphatase inhibitor-1 by cyclin-dependent kinase 5. The Journal of biological chemistry.

[26] Hashiguchi M, Saito T, Hisanaga S, Hashiguchi T (2002). Truncation of CDK5 activator p35 induces intensive phosphorylation of Ser202/Thr205 of human tau. The Journal of biological chemistry.

[27] Fu AK, Fu WY, Ng AK, Chien WW, Ng YP, Wang JH, Ip NY (2004). Cyclin-dependent kinase 5 phosphorylates signal transducer and activator of transcription 3 and regulates its transcriptional activity. Proceedings of the National Academy of Sciences of the United States of America.

[28] Lin H, Chen MC, Chiu CY, Song YM, Lin SY (2007). Cdk5 regulates STAT3 activation and cell proliferation in medullary thyroid carcinoma cells. The Journal of biological chemistry.

[29] Sherr CJ, Roberts JM (1999). CDK inhibitors: positive and negative regulators of G1-phase progression. Genes & development.

[30] Thullberg M, Bartkova J, Khan S, Hansen K, Ronnstrand L, Lukas J, Strauss M, Bartek J (2000). Distinct versus redundant properties among members of the INK4 family of cyclin-dependent kinase inhibitors. FEBS letters.

[31] Goretzki PE, Gorelov V, Dotzenrath C, Witte J, Roeher HD (1996). A frequent mutation/polymorphism in tumor suppressor gene INK4B (MTS-2) in papillary and medullary thyroid cancer. Surgery.

[32] van Veelen W, Klompmaker R, Gloerich M, van Gasteren CJ, Kalkhoven E, Berger R, Lips CJ, Medema RH, Hoppener JW, Acton DS (2009). P18 is a tumor suppressor gene involved in human medullary thyroid carcinoma and pheochromocytoma development. International journal of cancer Journal international du cancer.

[33] Flicker K, Ulz P, Hoger H, Zeitlhofer P, Haas OA, Behmel A, Buchinger W, Scheuba C, Niederle B, Pfragner R, Speicher MR (2012). High-resolution analysis of alterations in medullary thyroid carcinoma genomes. International journal of cancer Journal international du cancer.

[34] Franklin DS, Godfrey VL, O'Brien DA, Deng C, Xiong Y (2000). Functional collaboration between different cyclin-dependent kinase inhibitors suppresses tumor growth with distinct tissue specificity. Molecular and cellular biology.

[35] Joshi PP, Kulkarni MV, Yu BK, Smith KR, Norton DL, van Veelen W, Hoppener JW, Franklin DS (2007). Simultaneous downregulation of CDK inhibitors p18(Ink4c) and p27(Kip1) is required for MEN2A-RET-mediated mitogenesis. Oncogene.

[36] van Veelen W, van Gasteren CJ, Acton DS, Franklin DS, Berger R, Lips CJ, Hoppener JW (2008). Synergistic effect of oncogenic RET and loss of p18 on medullary thyroid carcinoma development. Cancer research.

[37] Gartel AL, Najmabadi F, Goufman E, Tyner AL (2000). A role for E2F1 in Ras activation of p21(WAF1/CIP1) transcription. Oncogene.

[38] Bracken AP, Ciro M, Cocito A, Helin K (2004). E2F target genes: unraveling the biology. Trends in biochemical sciences.

[39] Kurzrock R, Sherman SI, Ball DW, Forastiere AA, Cohen RB, Mehra R, Pfister DG, Cohen EE, Janisch L, Nauling F, Hong DS, Ng CS, Ye L, Gagel RF, Frye J, Muller T (2011). Activity of XL184 (Cabozantinib), an oral tyrosine kinase inhibitor, in patients with medullary thyroid cancer. Journal of clinical oncology : official journal of the American Society of Clinical Oncology.

[40] Plattner F, Angelo M, Giese KP (2006). The roles of cyclin-dependent kinase 5 and glycogen synthase kinase 3 in tau hyperphosphorylation. The Journal of biological chemistry.

[41] Livak KJ, Schmittgen TD (2001). Analysis of relative gene expression data using real-time quantitative PCR and the 2(−Delta Delta C(T)) Method. Methods.

[42] Pozo K, Cingolani LA, Bassani S, Laurent F, Passafaro M, Goda Y (2012). beta3 integrin interacts directly with GluA2 AMPA receptor subunit and regulates AMPA receptor expression in hippocampal neurons. Proceedings of the National Academy of Sciences of the United States of America.

[43] Bibb JA, Snyder GL, Nishi A, Yan Z, Meijer L, Fienberg AA, Tsai LH, Kwon YT, Girault JA, Czernik AJ, Huganir RL, Hemmings HC, Nairn AC, Greengard P (1999). Phosphorylation of DARPP-32 by Cdk5 modulates dopamine signalling in neurons. Nature.

