# Supplementary material for: Differential expression of cell cycle regulators in CDK5-dependent medullary thyroid carcinoma tumorigenesis
Source: Oncotarget. 2015 Apr 14;6(14):12080–93. doi: 10.18632/oncotarget.3813 (PMC4494924; doi:10.18632/oncotarget.3813)
Supplement: Supplementary file 1 [file oncotarget-06-12080-s001.pdf]

Differential expression of cell cycle regulators in CDK5-dependent medullary thyroid carcinoma tumorigenesis

Supplementary Material

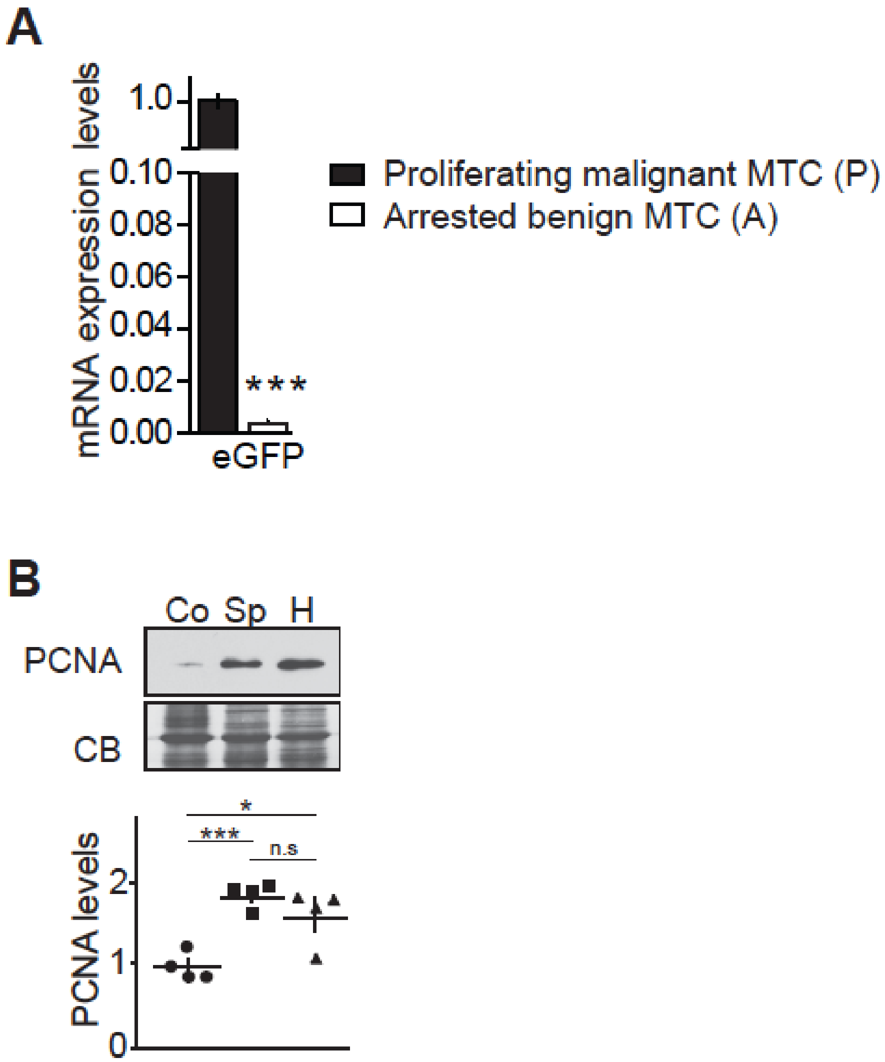

Figure S1. (**A**) RT-qPCR analysis of eGFP mRNA expression in proliferating malignant mouse MTC compared to arrested benign mouse MTCs; p-value < 0.0001 (N = 6 for each conditions. (**B**) Immunoblot of lysates from control, sporadic and hereditary human MTC probed with anti-PCNA antibodies. P-values were p = 0.2419 for Sp vs H, p = 0.0003 for Sp vs Co and p = 0.0223 for H vs Co. Data are represented as mean +/- SEM, N = 4 for each condition.

Table S1: A Table showing genes that are upregulated in proliferating malignant compared to arrested benign mouse MTCs. Genes that are mentioned in the main text are bolded.

GENE SYMBOL	LOG2-FOLD UPREGULATION	P-VALUE	GENE DESCRIPTION
eGFP	6.88	0.00006	
Troap	5.2	0.00000	trophinin associated protein
Pbk	5.1	0.00005	PDZ binding kinase
Spc25	4.95	0.00003	SPC25, NDC80 kinetochore complex component, homolog (S. cerevisiae)
LOC640739	4.91	0.00000	hypothetical protein LOC640739
2810417H13Rik	4.9	0.00034	predicted gene 15428; RIKEN cDNA 2810417H13 gene; predicted gene 9171
Kif18b	4.68	0.00070	kinesin family member 18B
**Birc5**	**4.63**	**0.00000**	**budding uninhibited by benzimidazoles 1 homolog, beta (S. cerevisiae)**
Clspn	4.62	0.00002	claspin homolog (Xenopus laevis)
Gtse1	4.44	0.00000	G two S phase expressed protein 1
Ckap2	4.43	0.00037	cytoskeleton associated protein 2
Crabp1	4.38	0.00024	cellular retinoic acid binding protein I
LOC100042970	4.31	0.00006	
Brrn1	4.29	0.00001	barren homolog (Drosophila)
Arhgef39	4.26	0.00056	Rho guanine nucleotide exchange factor (GEF) 39
**Cdc2a**	**4.22**	**0.00013**	**cyclin-dependent kinase 1**
**Plk1**	**4.18**	**0.00108**	**polo-like kinase 1 (Drosophila)**
**Rad51**	**4.18**	**0.00010**	**RAD51 homolog (S. cerevisiae)**
Cdca3	4.14	0.00028	cell division cycle associated 3
**Ccnb1**	**4.13**	**0.00008**	**cyclin B1**
Ncaph	4.12	0.00084	non-SMC condensin I complex, subunit H
Knstrn	4.06	0.00039	kinetochore-localized astrin/SPAG5 binding
**Aurkb**	**4.06**	**0.00114**	**aurora kinase B**
**Rrm2**	**4.05**	**0.00007**	**ribonucleotide reductase M2**
Cenpa	4.03	0.00076	centromere protein A
**Cdkn3**	**4.01**	**0.00043**	**cyclin-dependent kinase inhibitor 3**
LOC667005	4.01	0.00068	
Clhc1	4	0.00098	clathrin heavy chain linker domain containing 1
Kif4	3.91	0.00006	kinesin family member 4
Nusap1	3.85	0.00024	nucleolar and spindle associated protein 1
Spag5	3.85	0.00066	sperm associated antigen 5
Sgol1	3.83	0.00053	shugoshin-like 1 (S. pombe)

Uhrf1	3.83	0.00008	ubiquitin-like, containing PHD and RING finger domains, 1; predicted gene 5648; similar to nuclear zinc finger protein Np95
Top2a	3.78	0.00010	topoisomerase (DNA) II alpha
Cit	3.77	0.00055	citron
Prc1	3.77	0.00022	protein regulator of cytokinesis 1
Esco2	3.76	0.00048	establishment of cohesion 1 homolog 2 (S. cerevisiae)
**Mcm10**	**3.76**	**0.00000**	**minichromosome maintenance deficient 10 (S. cerevisiae)**
Cenpi	3.74	0.00003	centromere protein I
Kif15	3.66	0.00009	kinesin family member 15
Mki67	3.62	0.00013	antigen identified by monoclonal antibody Ki 67
Cdca8	3.56	0.00003	cell division cycle associated 8
C79407	3.56	0.00022	expressed sequence C79407
Cdc20	3.54	0.00005	cell division cycle 20 homolog (S. cerevisiae)
Hist1h2ag	3.53	0.00001	histone cluster 1, H2ad; histone cluster 1, H2ae; histone cluster 1, H2ag; histone cluster 1, H2ah; histone cluster 1, H2ai; similar to histone 2a; histone cluster 1, H2an; histone cluster 1, H2ao; histone cluster 1, H2ac; histone cluster 1, H2ab
**Ccne2**	**3.52**	**0.00062**	**cyclin E2**
Ckap2l	3.49	0.00010	cytoskeleton associated protein 2-like
**Cdc25c**	**3.45**	**0.00040**	**cell division cycle 25 homolog C (S. pombe)**
Kif23	3.42	0.00002	kinesin family member 23
Sapcd2	3.41	0.00005	suppressor APC domain containing 2
Iqgap3	3.39	0.00024	IQ motif containing GTPase activating protein 3
Cenpm	3.34	0.00002	centromere protein M
Kif11	3.32	0.00114	kinesin family member 11
Cd177	3.32	0.00078	CD177 antigen
Chek1	3.27	0.00005	checkpoint kinase 1 homolog (S. pombe)
Hist1h2ah	3.23	0.00006	histone cluster 1, H2ad; histone cluster 1, H2ae; histone cluster 1, H2ag; histone cluster 1, H2ah; histone cluster 1, H2ai; similar to histone 2a; histone cluster 1, H2an; histone cluster 1, H2ao; histone cluster 1, H2ac; histone cluster 1, H2ab
Sgol2	3.23	0.00002	shugoshin-like 2 (S. pombe)
Ncapg2	3.2	0.00009	non-SMC condensin II complex, subunit G2
Dmkn	3.19	0.00032	dermokine
Ska3	3.18	0.00049	spindle and kinetochore associated complex subunit 3
Hist1h2ak	3.17	0.00003	histone cluster 1, H2ak
Hist1h2ad	3.13	0.00024	histone cluster 1, H2ad; histone cluster 1, H2ae; histone cluster 1, H2ag; histone

			cluster 1, H2ah; histone cluster 1, H2ai; similar to histone 2a; histone cluster 1, H2an; histone cluster 1, H2ao; histone cluster 1, H2ac; histone cluster 1, H2ab
Rad54b	3.13	0.00101	RAD54 homolog B (S. cerevisiae)
Cenpn	3.12	0.00011	centromere protein N
**Ccna1**	**3.1**	**0.00058**	**cyclin A1**
2210011C24Rik	3.05	0.00000	RIKEN cDNA 2210011C24 gene
Depdc1b	3.05	0.00020	DEP domain containing 1B
**Hmgb2**	**3.05**	**0.00008**	**predicted gene 13160; predicted gene 8681; predicted gene 13237; predicted gene 4169; predicted gene 8284; similar to High mobility group box 2; predicted gene 13167; high mobility group box 2; predicted gene 13232**
Cenph	3.02	0.00033	centromere protein H
Kif18a	2.92	0.00077	kinesin family member 18A
Hist1h2af	2.87	0.00001	histone cluster 1, H2af
**Tcf19**	**2.86**	**0.00003**	**transcription factor 19**
Nrm	2.85	0.00010	nurim (nuclear envelope membrane protein)
Cep55	2.83	0.00033	centrosomal protein 55
**Hist2h2ab**	**2.82**	**0.00011**	**histone cluster 2, H2ab**
Hist1h2an	2.81	0.00010	histone cluster 1, H2ad; histone cluster 1, H2ae; histone cluster 1, H2ag; histone cluster 1, H2ah; histone cluster 1, H2ai; similar to histone 2a; histone cluster 1, H2an; histone cluster 1, H2ao; histone cluster 1, H2ac; histone cluster 1, H2ab
Oip5	2.78	0.00058	Opa interacting protein 5
BC004701	2.76	0.00029	baculoviral IAP repeat-containing 5
**Tyms**	**2.74**	**0.00019**	**thymidylate synthase**
Fbln1	2.7	0.00108	fibulin 1
**Rad51ap1**	**2.69**	**0.00076**	**RAD51 associated protein 1**
Chaf1b	2.68	0.00045	chromatin assembly factor 1, subunit B (p60)
Pif1	2.67	0.00015	PIF1 5'-to-3' DNA helicase homolog (S. cerevisiae)
Tmem91	2.61	0.00068	transmembrane protein 91
Gins1	2.6	0.00040	GINS complex subunit 1 (Psf1 homolog)
Bub1b	2.54	0.00023	budding uninhibited by benzimidazoles 1 homolog, beta (S. cerevisiae)
Fam132b	2.53	0.00101	family with sequence similarity 132, member B
Rad54l	2.47	0.00064	RAD54 like (S. cerevisiae)
Hist1h2ai	2.44	0.00012	histone cluster 1, H2ad; histone cluster 1, H2ae; histone cluster 1, H2ag; histone cluster 1, H2ah; histone cluster 1, H2ai; similar to histone 2a; histone cluster 1, H2an; histone cluster 1, H2ao; histone

			cluster 1, H2ac; histone cluster 1, H2ab
Tyms-ps	2.44	0.00006	thymidylate synthase, pseudogene
Ifi27	2.43	0.00050	interferon, alpha-inducible protein 27
**Melk**	**2.42**	**0.00005**	**maternal embryonic leucine zipper kinase**
Psrc1	2.42	0.00063	proline/serine-rich coiled-coil 1
B230323D24Rik	2.39	0.00073	
Phf19	2.36	0.00014	PHD finger protein 19
Cthrc1	2.35	0.00061	collagen triple helix repeat containing 1
Cenpw	2.33	0.00007	centromere protein W
**Recql4**	**2.31**	**0.00011**	**RecQ protein-like 4**
Pkmyt1	2.28	0.00029	protein kinase, membrane associated tyrosine/threonine 1
scl0002507.1_236	2.26	0.00044	
Kcne2	2.25	0.00009	potassium voltage-gated channel, Isk-related subfamily, gene 2
Gas2l3	2.24	0.00012	similar to growth arrest-specific 2 like 3; growth arrest-specific 2 like 3
Slc4a1	2.22	0.00053	solute carrier family 4 (anion exchanger), member 1
Ccr5	2.21	0.00008	chemokine (C-C motif) receptor 5
**Pcna**	**2.21**	**0.00071**	**proliferating cell nuclear antigen; similar to proliferating cell nuclear antigen (DNA polymerase delta auxiliary protein)**
Dbf4	2.2	0.00050	DBF4 homolog (S. cerevisiae)
**Fam111a**	**2.19**	**0.00001**	**family with sequence similarity 111, member A**
**Tmpo**	**2.16**	**0.00033**	**thymopoietin**
Mad2l1	2.15	0.00014	similar to spindle assembly checkpoint protein; MAD2 mitotic arrest deficient-like 1 (yeast)
Spink4	2.14	0.00033	serine peptidase inhibitor, Kazal type 4
Smc4	2.13	0.00003	structural maintenance of chromosomes 4
Fabp5	2.12	0.00043	fatty acid binding protein 5, epidermal
4930427A07Rik	2.11	0.00048	RIKEN cDNA 4930427A07 gene
LOC245892	2.1	0.00037	
Arpp21	2.06	0.00027	cyclic AMP-regulated phosphoprotein, 21
Ddx11	2.03	0.00109	DEAD/H (Asp-Glu-Ala-Asp/His) box polypeptide 11 (CHL1-like helicase homolog, S. cerevisiae)

Table S2: A Table showing downregulated genes in proliferating malignant compared to arrested benign mouse MTCs

GENE SYMBOL	LOG2-FOLD UPREGULATION	P-VALUE	GENE DESCRIPTION
Epb4.1l5	-2.54	0.001	erythrocyte protein band 4.1-like 5
KIF17	-2.45	0.001	kinesin family member 17
Leprel1	-2.23	0.001	leprecan-like 1
Lgals4	-2.21	0.000	lectin, galactose binding, soluble 4
LOC385274	-2.19	0.001	
Luzp2	-2.36	0.001	leucine zipper protein 2
Mtap2	-3.52	0.000	microtubule-associated protein 2

Supplemental methods: analysis of somatic RET mutation

Genomic DNA was isolated from dissected sections containing at least 80% of neoplastic cells using the QIAamp DNA FFPE tissue kit (QIAGEN, Valencia, CA) according to the manufacturer's instructions. DNA was amplified by PCR using primers flanking known mutational hotspots (see Table below). PCR reactions were performed in 25 µL and consisted of: 1× PCR buffer, 250 ng of genomic DNA, 0.4 µmol/L each primer, 0.1 mmol/L each deoxynucleoside triphosphates, 0.75 unit of TaKaRa LA Taq DNA Polymerase (TaKaRa Bio USA, Madison, WI). After an initial cycle of denaturation at 94°C for 3 minutes, amplifications were performed as follows: 40 cycles of denaturation at 94°C for 45 seconds, annealing at 58°C for 30 seconds, and extension at 72°C for 90 seconds with and final extension at 72°C for 10 minutes. The PCR products were cleaned up with ExoSAP-IT (USB, Cleveland, OH) according to manufacturer instructions and sent to the core facility for sequencing using nested primers.

EXON	FORWARD PRIMER SEQUENCE (5'-3')	REVERSE PRIMER SEQUENCE (5'-3')	SEQUENCING PRIMER (5'-3')
10	GGCTAAGCCAAGCTGCTG	CCATGAAACTTCCCTCCCTC	TATGCTTGCGACACCAGTTG
11	GTTCTCAGGCCTTCCCACAC	TGCGGGGCAGGGAAGATCCCC	ACACCTCCATGGCCACTTC
13	CTCTCTGTCTGAACTTGGGC	CTACTGCTCCTGCCCTGTT	AGGTCCATCCTGACCTGGTA
14	TCCACCCCCTTACTCATTGG	GGAAAGATACCGAAGATTAGTG	GCATGGTGGGCTAGAGTGTG
15	TCCACCCCCTTACTCATTGG	GGAAAGATACCGAAGATTAGTG	CATGTCACACCCTGACTCCA
16	ACTCCCGCCAGCATCTCAG	GTTCGTGAGGCAAATGGCT	GCCCCTTCAAAGATGTGTGT

Exons 14 and 15 were amplified as a single PCR product

Table S3: Primers used for the RT-qPCR analyses

GENE NAME	GENE SYMBOL	ACCESSION NO	PRIMER SEQUENCE 5' - 3'	AMPLICON (BP)
	eGFP		F: ATCATGGCCGACAAGCAGAAGAAC<br>R: GTACAGCTCGTCCATGCCGAGAGT	257
	B2m	NM009735	F: TGCTATCCAGAAAACCCCTCAA<br>R: CAATGTGAGGCGGGTGGAA	110
p15INK4b	cdkn2b	NM007670	F: AGCTGGATCTGGTCCTTGAG<br>R: GATCCAAGAATTTCCCTTGC	130
p16INK4a	cdkn2a	NM009877	F: CAAGAGCGGGGACATCAAGA<br>R: TTGAGCTGAAGCTATGCCCG	127
p18INK4c	cdkn2c	NM007671	F: GGCTGTCCGTTTCACTAT CA<br>R: TTTTGAAGGATTTGGCTGCT	85
p19INK4d	cdkn2d	NM009878	F: TTGAAGAAGGGAGTGGGAGGA<br>R: ACCGTTTAGATGGCTGTTGC	153
p21CIP/WAF1	cdkn1a	NM007669	F: GTACTTCCTCTGCCCTGCTG<br>R: TCTGCGCTTGGAGTGATAGA	173
p27KIP	cdkn1b	NM009875	F: GTCCAGGGATGAGGAAGCGAC<br>R: CAGTGCCAGCGTTCGGG	108
cyclin A1	ccna1	NM007628	F: ATCCTGAACAGGGGGACAGA<br>R: GCATTGGGGAAACTGTGTTGA	135
cyclin B1	ccnb1	NM172301	F: GCTCTCCATGCTGGACTACG<br>R: TAGCCAGGTGCTGCATAACA	170
cyclin D1	ccnd1	NM007631	F: GCGTACCCTGACACCAATCT<br>R: CACAGACCTCCAGCATCCAG	160
cyclin E1	ccne1	NM007633	F: GGCAAATGTGGCCGTGTTTT<br>R: GGATGAAAGAGCAGGGGTCC	131
cyclin E2	ccne2	NM001037134	F: AGAGAAAAACAGCACAGGATGTC<br>R: ATGCAAGGGCTGATTCCTCC	115
p35	cdk5r1	NM009871	F: TGGCTTGGGGTTTGGAAGTG<br>R: GGAAACCCTCCGCAGTATCTT	110
Cdk1	cdk1	NM007659	F: GTCCGTCGTAACCTGTTGAGT<br>R: ACCACACCGTAAGTACCTTCTC	78
Cdk2	cdk2	NM016756	F: CTGGGCTGCAAGTACTACTCC<br>R: CCTGCGGGTCACCATTTCA	78
Cdk4	cdk4	NM009870	F: GACCGATCCCCGGTGTATG<br>R: GAAGCAGGGGATCTTACGCT	98
Cdk5	cdk5	NM007668	F: ACCCAGCTACAACATCCTTGG<br>R: CGCTGCACAGGGTTACACTT	97
Cdk6	cdk6	NM009873	F: ACCCACAGAAACCATAAAGGATA<br>R: GCGGTTTCAGATCACGATGC	95
